# Supplementary material for: Enhanced Metabolome Coverage and Evaluation of Matrix Effects by the Use of Experimental-Condition-Matched 13C-Labeled Biological Samples in Isotope-Assisted LC-HRMS Metabolomics
Source: Metabolites. 2020 Oct 27;10(11):434. doi: 10.3390/metabo10110434 (PMC7692853; doi:10.3390/metabo10110434)
Supplement: Supplementary file 1 [file metabolites-10-00434-s001.pdf]

Supporting Information

Enhanced Metabolome Coverage and Evaluation of Matrix Effects by the Use of Experimental-Condition-Matched ^{13}C-Labeled Biological Samples in Isotope-Assisted LC-HRMS Metabolomics

Asja Ćeranić [1,†], Christoph Bueschl [1,†], Maria Doppler [1], Alexandra Parich [1], Kangkang Xu [1], Marc Lemmens [2], Hermann Buerstmayr [2] and Rainer Schuhmacher [1,*]

[1] Institute of Bioanalytics and Agro-Metabolomics, Department of Agrobiotechnology, IFA-Tulln, University of Natural Resources and Life Sciences Vienna (BOKU), Konrad-Lorenz-Strasse 20, Tulln an der Donau, 3430, Upper Austria, Austria; asja.ceranic@boku.ac.at (A.C.); christoph.bueschl@boku.ac.at (C.B.); maria.doppler@boku.ac.at (M.D.); alexandra.parich@boku.ac.at (A.P.); kangkang.xu@boku.ac.at (K.X.)

[2] Institute of Biotechnology in Plant Production, Department of Agrobiotechnology, IFA-Tulln, University of Natural Resources and Life Sciences Vienna (BOKU), Konrad-Lorenz-Strasse 20, Tulln an der Donau, 3430, Upper Austria, Austria; marc.lemmens@boku.ac.at (M.L.); hermann.buerstmayr@boku.ac.at (H.B.)

* Correspondence: rainer.schuhmacher@boku.ac.at; Tel.: +43-1-47654-97307

† These authors contribute equally to the work

Received: 20 September 2020; Accepted: 22 October 2020; Published: date

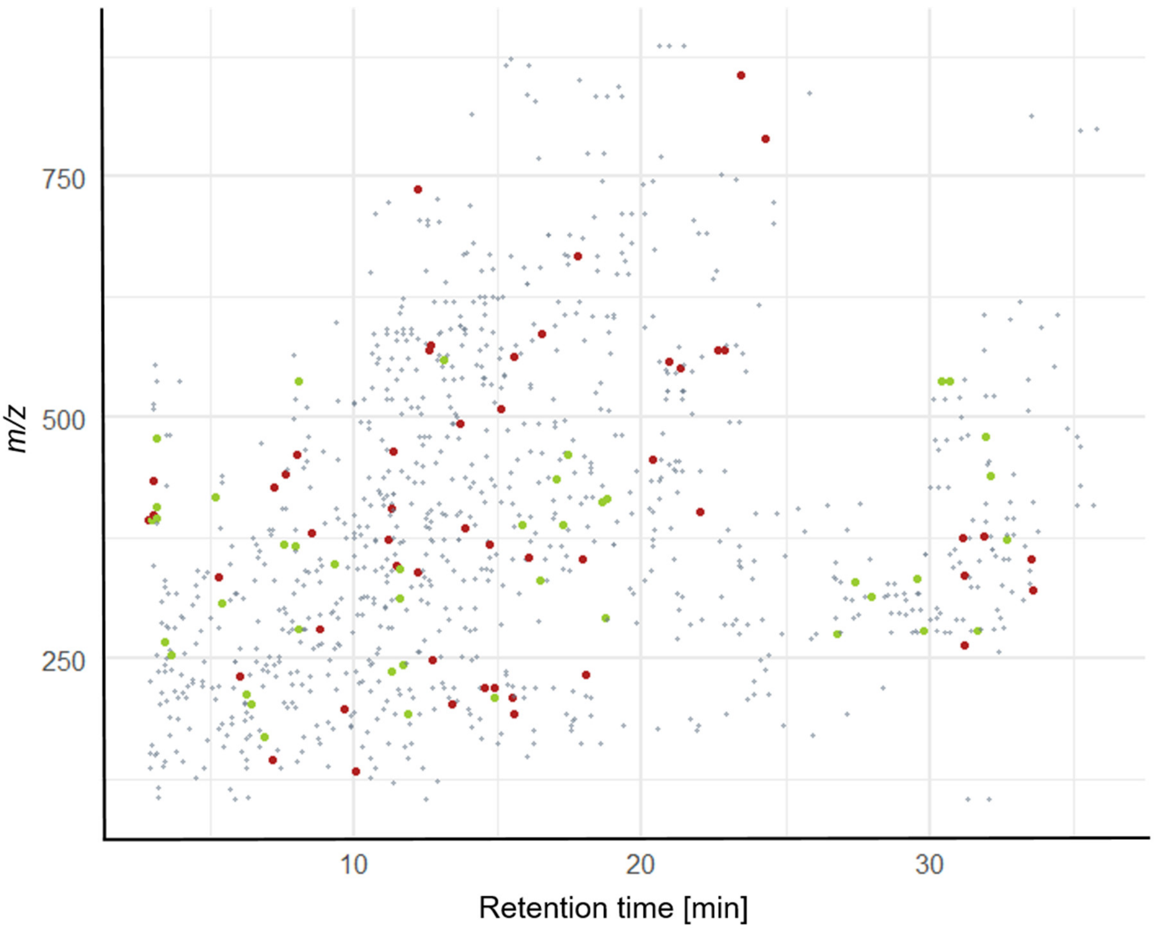

Figure S1. Feature map of detected metabolites. Different color code represents metabolites that have shown different results for the significantly differing metabolites in the comparative quantification test (Toxin versus Control). In red are highlighted those which are significantly different (between

Toxin and Control) only if absolute native abundances are used, and in green only those obtained after normalized (native to labeled) abundances are applied.
